# Infection Rates in Open Fractures of the Tibia: Is the 6-Hour Rule Fact or Fiction?

**DOI:** 10.4061/2011/943495

**Published:** 2011-10-27

**Authors:** Ameya S. Kamat

**Affiliations:** Department of Orthopaedic Surgery, Wellington Public Hospital, 8D/39 Taranaki Street, Te Aro, Wellington 6011, New Zealand

## Abstract

*Aims*. Emergency debridement has long been the standard of care for open fractures of the tibia as infection is an important complication. The timing of operative debridement can be debated. We review open fractures of the tibia and compare infection rates in those that were operated on within and after 6-hours. *Method*. 103 consecutive open fractures of the tibia were reviewed. The data was analysed retrospectively with regard to severity of fracture and incidence of infection. Infection rates over a three-month period were compared between the two groups. *Results*. 12 (11.6%) patients developed an infection within the first 3 months of injury. 7 of which were taken to theatre within 6-hours, and 5 after 6-hours. No significant differences were found between these two groups. *Conclusion*. There is no significant difference in timing of surgery. Initial basic interventions may play more of a role in limiting the risk of infection.

## 1. Introduction

Emergency operative measures have long been the standard of care for open fractures of the tibia as deep infection is the most important complication. While there is unanimous agreement with regards to early operative debridement of wounds, there have been only a few articles reflecting timing [1, 2]. Debridement of the open wound within six hours after the injury is a widely accepted standard of care [3].

The precise origins of the so-called “six-hour rule” are unclear. Some claim that it stems from an 1898 experiment during the Spanish-American war by German military surgeon Friedrich [4], in which guinea pigs with contaminated soft-tissue wounds had lower rates of infection when debridement was performed within six hours. Others however point to a 1973 study by Robson et al., who reported that 105 organisms per gram of tissue was the open-fracture infection threshold, which was reached in an average of 5.17 hours [5].

There have been credible articles to date showing evidence that the 6-hour rule should not be cast in stone [6].

This paper reviews open fractures of the tibia and compares infection rates in those that were operated on within 6-hours and those operated on after 6-hours.

## 2. Methods

103 consecutive open fractures of the tibia were reviewed amongst hospitals in the Wellington region of New Zealand over the last 10 years. 

Patients were included in the study if they were above the age of 16 (skeletally mature patients) and had presented to one of the three participating hospitals for treatment of an open fracture to the tibia. Patients with intra-articular fractures of the tibia were excluded. Patients were also excluded if they had life-threatening head, chest, or abdominal injuries as these would take priority over any limb-threatening injury. Patients with documented mental illness and third-degree burns completed our exclusion criteria.

The study required the patient to be examined by the on-call orthopaedic surgeon or registrar/resident.

The data obtained from patients records was analysed retrospectively with regard to severity of the fracture (using classification system of Gustilo and Anderson) and incidence of infection over a three-month period [7].

Gustilo et al. classified open fractures into three categories: [7, 8].

- Grade (Type) I: open fracture with a skin wound less than 1 cm long and clean,
- Grade (Type) II: open fracture with a laceration more than 1 cm long without extensive soft tissue damage, flaps, or avulsions,
- Grade (Type) III: either an open segmental fracture, an open fracture with extensive soft tissue damage, or a traumatic amputation.
- Gustilo stated that Type III open fractures were too complicated and hence further stratified these wounds:
- IIIa: adequate soft tissue coverage of a fractured bone despite extensive soft tissue lacerations or flaps, or high energy trauma irrespective of the size of the wound. This includes segmental fractures or severely comminuted fractures;
- IIIb: extensive soft tissue injury loss with periosteal stripping and bone exposure. This is usually associated with massive contamination;
- IIIc: open fractures associated with vascular injury requiring repair for limb salvage.

We treated open fractures of the tibia in the following manner:

1. early administration of intravenous broad-spectrum antibiotics, either 2 g of Cefuroxime or 2 g of Cephazolin three times a day for a minimum of 48 hours, followed by 1 g of oral Flucloxacillin four times a day for seven days after discharge [9],
2. wound irrigation in the emergency department as the patient was awaiting theatre,
3. intraoperative wound debridement and thorough irrigation—3 litres of normal saline per Gustilo grade,
4. if possible, primary closure of the wound,
5. secondary closure in heavily contaminated wounds,
6. fixation comprised of either internal fixation, external fixation or cast immobilization and was at the absolute discretion of the surgeon,
7. analgesia and intravenous fluids on an as-required basis,
8. dressings/casting as per type of fixation/immobilization.

After discharge, patients were followed-up in the outpatient fracture clinic for wound review and suture removal at the 10-day mark.

Infection was documented irrespective of the type of closure or fixation and irrespective of culture results. The spectrum of infection would include

1. cellulitis,
2. wound breakdown,
3. stitch abscess,
4. purulent discharge or ooze,
5. established collection/abscess,
6. infected metalware where applicable,
7. osteomyelitis.

Patients were divided into two groups. One being those who were taken to theatre within 6-hours, and the other consisting of patients taken to theatre after 6-hours. 

The infection rate was determined by the number of patients infected divided by the number of patients in each group.

## 3. Results

103 consecutive open fractures of the tibia were reviewed. 62 patients were taken to theatre for surgical debridement within 6-hours and 41 after 6-hours.

There were 49 patients with Grade I fractures, of which, 19 were operated upon during the first 6 hours, and 30 were operated on after 6 hours. 

**Figure 1:**
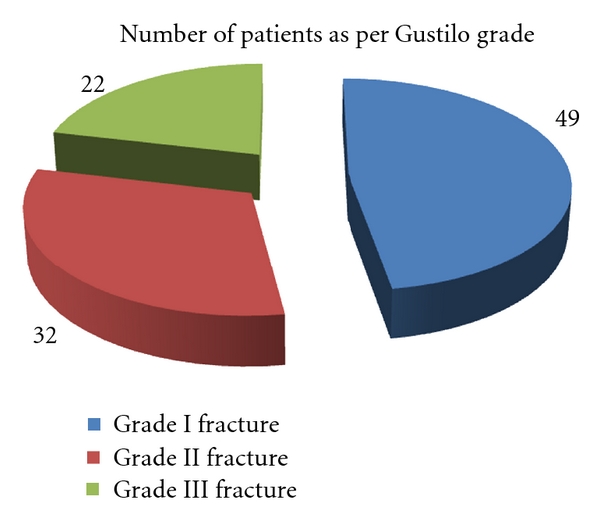
Fractures as per Gustilo grade.

There were 32 patients with Grade II fractures, of which, 21 were operated upon within 6 hours, and 11 were operated on after 6 hours. There were 22 patients with Grade III fractures, of which, 12 were operated upon within 6 hours, and 10 were operated on after 6 hours (See Figure 1).

With regards to timing, the mean time to theatre in the “within 6-hour group” was 3.25 hours. The range for this was 1.25 to 5.15 hours. The mean time to theatre in the “post 6-hour group” was 9.15 hours. The range for this group was 6.15 to 17.25 hours.

**Figure 2:**
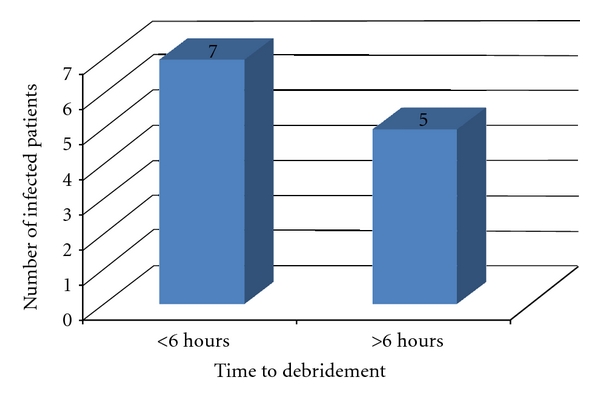
The infection rate amongst the two groups.

12 out of the 103 cases sustained infections, that is, an 11.6% infection rate. 7 of which were taken to theatre within 6-hours, and 5 after 6-hours (See Figure 2).

**Figure 3:**
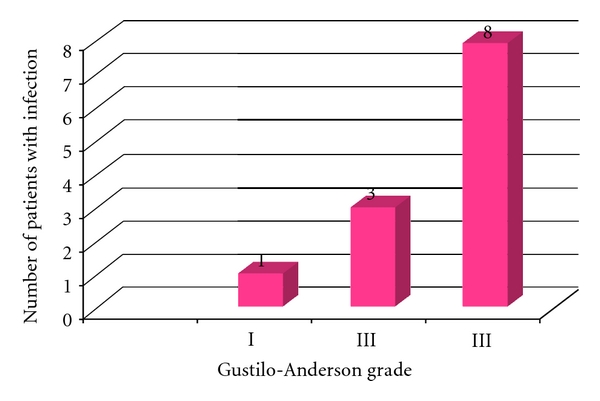
Number of patients who developed infection within each Gustilo grade.

From these 12 cases, 1 was a Grade I fracture (8.3%), 3 were Grade II fractures (25%), and the remaining 8 were Grade III fractures (66.6%) (See Figure 3). 

With regard to Grade III fractures, these were further subclassified into Grade IIIA and Grade IIIB, and each of these grades had 4 patients who sustained infections.

For the patients who sustained infections, a breakdown with regard to individual time to debridement is included in the table (See Table 1).

From the 12 cases, 6 patients sustained cellulitis around the wound edges, 3 suffered from wound break down, 1 patient had a formal pus-filled collection, and 2 sustained osteomyelitis.

The Infection rate of patients taken to theatre within 6-hours was 11% whereas that of those operated on after 6-hours was 12.1%—*P* > 0.05, showing no statistical difference There was also no statistical significance when comparing patients with infection in the Grade IIIA and IIIB categories, nor was there any significance related to fracture comminution.

From the 12 patients who sustained infections, 10 had no previous significant medical illnesses such as diabetes, obesity, or hypertension. Only one patient was a smoker from the patients who developed infections.

As mentioned previously, the definitive fixation was at the discretion of the surgeon and comprised of either casting, internal fixation, intramedullary devices or external fixators. There was no statistical relation to infection amongst the different treatment strategies.

## 4. Conclusion

The data gathered over the last decade indicates that there was no significant difference in terms of timing of surgery. 

As mentioned above, only a few studies have questioned the validity of this so-called 6-hour rule, mentioning that there is suggestion that infection rates are not dependent on timing of surgery [2, 9]. One of these studies [2] however had their data set comprising of fractures of both the tibia and femur, whereas this study concentrates on tibial fractures alone. 

However, on the opposing side, there have been two notable studies that are strong advocates of early operative debridement of open fractures claiming that infection rates can be lowered [10, 11]. 

Open fractures of the tibia do represent a challenge to even the most highly experienced orthopaedic surgeon. It is widely accepted that antibiotics should be administered as soon as possible. Early operative debridement remains important although there is limited evidence in support of an actual “six-hour rule.” Copious irrigation in the emergency department is useful as well.

There are numerous reasons why I believe that this study is significantly different to others. Firstly it focuses solely on open fractures of the tibia in adults whereas others do not. It has the largest data set when compared to other studies. Also, there is no statistical difference to the outcome of infection when measuring the method of fixation and patient co-morbidities. 

There are numerous pitfalls and limitations in this study however. The type of skin closure, level of contamination, surgeon discretion, other life-threatening head and chest or abdominal injuries. Patient comorbidities and type of fixation did not prove statistically significant in this data set. It was unfortunate that all these confounders could not be tested as they would result in too few numbers and hence statistical analysis would be unreliable.

It would also not be ethical to perform a randomised control trial to determine infection rates after open fractures of the tibia. 

Given the above, there cannot be a hard and fast rule. However, it is apparent that initial basic interventions such as wound irrigation in the emergency department, sterile antiseptic dressings, and most importantly, administration of intravenous broad-spectrum antibiotics play a crucial role in infection prevention of open fractures of the tibia. Timing of surgery itself may not be as crucial.

## Figures and Tables

**Table 1:** Stratification of infected cases stating distribution with regard to Gustilo-Anderson classification and time to debridement of each individual case.

Infected case	Gustilo-Anderson Grade	Time to debridement (hours)
(1)	I	14.5
(2)	II	9.25
(3)	II	12.5
(4)	II	1.15
(5)	IIIA	3.45
(6)	IIIA	5.45
(7)	IIIA	5.55
(8)	IIIA	3.25
(9)	IIIB	9.25
(10)	IIIB	1.00
(11)	IIIB	8.45
(12)	IIIB	2.15

## References

[1] Werner CM, Pierpont Y, Pollak AN (2008). The urgency of surgical débridement in the management of open fractures. *The Journal of the American Academy of Orthopaedic Surgeons*.

[2] Bednar DA, Parikh J (1993). Effect of time delay from injury to primary management on the incidence of deep infection after open fractures of the lower extremities caused by blunt trauma in adults. *Journal of Orthopaedic Trauma*.

[3] Pollak AN (2006). Timing of débridement of open fractures. *The Journal of the American Academy of Orthopaedic Surgeons*.

[4] Friedrich PL (1898). Die aseptische Versorgung frischer Wundern. *Langenbecks Archiv fur Klinische Chirurgie*.

[5] Robson MC, Duke WF, Krizek TJ (1973). Rapid bacterial screening in the treatment of civilian wounds. *Journal of Surgical Research*.

[6] Pollak N, Jones AL, Castillo RC, Bosse MJ, MacKenzie EJ (2010). The relationship between time to surgical débridement and incidence of infection after open high-energy lower extremity trauma. *Journal of Bone and Joint Surgery*.

[7] Gustilo RB, Anderson JT (1976). Prevention of infection in the treatment of one thousand and twenty five open fractures of long bones: retrospective and prospective analyses. *Journal of Bone and Joint Surgery*.

[8] Gustilo RB, Mendoza RM, Williams DN (1984). Problems in the management of type III (severe) open fractures: a new classification of type III open fractures. *Journal of Trauma*.

[9] Ashford RU, Mehta JA, Cripps R (2004). Delayed presentation is no barrier to satisfactory outcome in the management of open tibial fractures. *Injury*.

[10] Kindsfater K, Jonassen EA (1995). Osteomyelitis in grade II and III open tibia fractures with late debridement. *Journal of Orthopaedic Trauma*.

[11] Kreder HJ, Armstrong P (1995). A review of open tibia fractures in children. *Journal of Pediatric Orthopaedics*.

